# Frontoparietal network integrity supports cognitive function in pre‐symptomatic frontotemporal dementia: Multimodal analysis of brain function, structure, and perfusion

**DOI:** 10.1002/alz.14299

**Published:** 2024-10-17

**Authors:** Xulin Liu, Peter Simon Jones, Maurice Pasternak, Mario Masellis, Arabella Bouzigues, Lucy L. Russell, Phoebe H. Foster, Eve Ferry‐Bolder, John van Swieten, Lize Jiskoot, Harro Seelaar, Raquel Sanchez‐Valle, Robert Laforce, Caroline Graff, Daniela Galimberti, Rik Vandenberghe, Alexandre de Mendonça, Pietro Tiraboschi, Isabel Santana, Alexander Gerhard, Johannes Levin, Sandro Sorbi, Markus Otto, Florence Pasquier, Simon Ducharme, Chris Butler, Isabelle Le Ber, Elizabeth Finger, Maria Carmela Tartaglia, Matthis Synofzik, Fermin Moreno, Barbara Borroni, Jonathan D. Rohrer, Kamen A. Tsvetanov, James B. Rowe, The GENFI consortium

**Affiliations:** ^1^ Department of Clinical Neurosciences and Cambridge University Hospitals NHS Trust University of Cambridge Cambridge UK; ^2^ Sunnybrook Health Sciences Centre Sunnybrook Research Institute Toronto Canada; ^3^ University of Toronto Toronto Canada; ^4^ Dementia Research Centre Department of Neurodegenerative Disease UCL Institute of Neurology Queen Square London UK; ^5^ Department of Neurology Erasmus Medical Centre Rotterdam The Netherlands; ^6^ Alzheimer's disease and Other Cognitive Disorders Unit Neurology Service Hospital Clínic Institut d'Investigacións Biomèdiques August Pi I Sunyer University of Barcelona Barcelona Spain; ^7^ Clinique Interdisciplinaire de Mémoire Département des Sciences Neurologiques CHU de Québec, and Faculté de Médecine, Université Laval Québec Canada; ^8^ Karolinska Institute Department NVS, Centre for Alzheimer Research Division of Neurogenetics Stockholm Sweden; ^9^ Unit for Hereditary Dementias Theme Aging, Karolinska University Hospital Solna Stockholm Sweden; ^10^ Fondazione IRCCS Ospedale Policlinico Milan Italy; ^11^ Centro Dino Ferrari University of Milan Milan Italy; ^12^ Laboratory for Cognitive Neurology Department of Neurosciences KU Leuven Leuven Belgium; ^13^ Neurology Service University Hospitals Leuven Leuven Belgium; ^14^ Faculty of Medicine University of Lisbon Lisbon Portugal; ^15^ Fondazione IRCCS Istituto Neurologico Carlo Besta Milano Italy; ^16^ Faculty of Medicine University of Coimbra Coimbra Portugal; ^17^ Centre of Neurosciences and Cell Biology University of Coimbra Coimbra Portugal; ^18^ Division of Psychology Communication and Human Neuroscience Wolfson Molecular Imaging Centre University of Manchester First floor, Core Technology Facility Manchester UK; ^19^ Department of Nuclear Medicine Centre for Translational Neuro‐ and Behavioral Sciences University Medicine Essen Essen Germany; ^20^ Department of Geriatric Medicine Klinikum Hochsauerland Arnsberg Germany; ^21^ Department of Neurology Ludwig‐Maximilians Universität München Munich Germany; ^22^ Centre for Neurodegenerative Diseases (DZNE) Munich Germany; ^23^ Munich Cluster of Systems Neurology Munich Germany; ^24^ Department of Neurofarba University of Florence Firenze Italy; ^25^ IRCCS Fondazione Don Carlo Gnocchi Florence Firenze Italy; ^26^ Department of Neurology University of Ulm Ulm Germany; ^27^ University Lille Lille France; ^28^ Inserm 1172 Lille France; ^29^ CHU, CNR‐MAJ, Labex Distalz, LiCEND Lille Lille France; ^30^ Department of Psychiatry McGill University Health Centre McGill University Montreal Canada; ^31^ McConnell Brain Imaging Centre Montreal Neurological Institute McGill University Montreal Canada; ^32^ Nuffield Department of Clinical Neurosciences Medical Sciences Division University of Oxford Oxford UK; ^33^ Department of Brain Sciences Imperial College London Burlington Danes The Hammersmith Hospital London UK; ^34^ Paris Brain Institute – Institut du Cerveau – ICM Sorbonne Université Inserm U1127, CNRS UMR 7225, AP‐HP – Hôpital Pitié‐Salpêtrière Paris France; ^35^ Reference center for rare or early‐onset dementias IM2A Department of Neurology AP‐HP – Pitié‐Salpêtrière Hospital Paris France; ^36^ Department of Neurology AP‐HP – Pitié‐Salpêtrière Hospital Paris France; ^37^ Department of Clinical Neurological Sciences University of Western Ontario London Canada; ^38^ Tanz Centre for Research in Neurodegenerative Disease Toronto Western Hospital Toronto Ontario Canada; ^39^ Department of Neurodegenerative Diseases Hertie‐Institute for Clinical Brain Research & Centre of Neurology University of Tübingen Tübingen Germany; ^40^ Centre for Neurodegenerative Diseases (DZNE) Tübingen Germany; ^41^ Cognitive Disorders Unit Department of Neurology Hospital Universitario Donostia San Sebastian Gipuzkoa Spain; ^42^ Neuroscience Area Biodonostia Health Research Institute San Sebastian Gipuzkoa Spain; ^43^ Neurology Unit Department of Clinical and Experimental Sciences University of Brescia Brescia Italy; ^44^ Department of Psychology University of Cambridge Cambridge UK; ^45^ MRC Cognition and Brain Science Unit University of Cambridge Cambridge UK

**Keywords:** atrophy, cerebral blood flow, frontotemporal dementia, functional network, multimodal neuroimaging, pre‐symptomatic dementia

## Abstract

**INTRODUCTION:**

Genetic mutation carriers of frontotemporal dementia can remain cognitively well despite neurodegeneration. A better understanding of brain structural, perfusion, and functional patterns in the pre‐symptomatic stage could inform accurate staging and potential mechanisms.

**METHODS:**

We included 207 pre‐symptomatic genetic mutation carriers and 188 relatives without mutations. The gray matter volume, cerebral perfusion, and resting‐state functional network maps were co‐analyzed using linked independent component analysis (LICA). Multiple regression analysis was used to investigate the relationship of LICA components to genetic status and cognition.

**RESULTS:**

Pre‐symptomatic mutation carriers showed an age‐related decrease in the left frontoparietal network integrity, while non‐carriers did not. Executive functions of mutation carriers became dependent on the left frontoparietal network integrity in older age.

**DISCUSSION:**

The frontoparietal network integrity of pre‐symptomatic mutation carriers showed a distinctive relationship to age and cognition compared to non‐carriers, suggesting a contribution of the network integrity to brain resilience.

**Highlights:**

- A multimodal analysis of structure, perfusion, and functional networks.
- The frontoparietal network integrity decreases with age in pre‐symptomatic carriers only.
- Executive functions of pre‐symptomatic carriers dissociated from non‐carriers.

## 1 BACKGROUND

Frontotemporal dementia (FTD) is characterized by the selective degeneration of the frontal and temporal cortices, leading to progressive deficits in behavior, social and executive function, or language.^1^ Genetic risk factors are important, with about 20%–30% of FTD cases being familial.^2^ Highly penetrant mutations in three major genes, chromosome 9 open reading frame 72 (*C9orf72*), microtubule‐associated protein tau (*MAPT*), and progranulin (*GRN*), account for about 60% of cases of familial FTD.^1^ Given that neurobiological changes could occur many years before the onset of symptoms of neurodegenerative dementias,^3^, ^4^, ^5^, ^6^ investigation at the early stage of diseases before symptom onset is important for understanding factors that facilitate the brain's resilience. Genetic FTD with highly penetrant genetic mutations provides the opportunity for early investigation before symptom onset. Comparison between pre‐symptomatic genetic mutation carriers and their family members without the mutation, allows one to investigate the effect of early neurodegeneration without the confounding influence of medication and lifestyle changes after symptom onset.

People carrying highly penetrant genetic mutations of FTD have gray matter atrophy and a reduction in cerebral blood flow (CBF) more than a decade before the expected symptom onset, as measured by magnetic resonance imaging (MRI) and arterial spin labeling (ASL).^4^, ^6^, ^7^, ^8^ However, functional network organization and connectivity are generally maintained despite significant atrophy in pre‐symptomatic genetic FTD.^4^, ^9^ Moreover, a recent study indicates that functional networks predict cognitive decline and symptomatic conversion in pre‐symptomatic genetic mutation carriers.^10^ A better understanding of these changes in the pre‐symptomatic stage would inform accurate staging, facilitate clinical trials, and elucidate the mechanisms of resilience by which gene carriers remain cognitively well for many years despite biomarker evidence of neurodegeneration.

Here, we test whether pre‐symptomatic differences in brain structure, cerebral perfusion, and functional network act synergistically or independently on clinically relevant disease features such as cognitive performance, and disease progression. Specifically, we used linked independent components analysis of multimodal imaging to investigate whether the interplay of brain gray matter atrophy, cerebral perfusion, and functional network integrity explains difference between pre‐symptomatic FTD genetic mutation carriers and non‐carriers.

**RESEARCH IN CONTEXT**

1. **Systematic review**: The authors systematically reviewed the literature using PubMed, preprint repositories, and research citing key articles. The alternations of brain structure, function, and perfusion have been characterized at the pre‐symptomatic stage of frontotemporal dementia in literature but are often studied separately. The inter‐correlated effects of brain structure, function, and perfusion in relation to genetic mutation status and cognition are not well‐characterized at the pre‐symptomatic stage.
2. **Interpretation**: Our results suggest that the frontoparietal network integrity of pre‐symptomatic carriers showed a distinctive relationship to age and cognitive functions compared to non‐carriers, despite age‐related atrophy and hypoperfusion. Functional network integrity may contribute to brain resilience in pre‐symptomatic frontotemporal dementia, mitigating the effects of atrophy and hypoperfusion in the late pre‐symptomatic stage.
3. **Future directions**: These results would inform possible ways to delay symptom onset by maintaining functional network integrity.

## 2 METHODS

### 2.1 Participants

The Genetic Frontotemporal dementia Initiative (GENFI) study is an international muti‐center cohort study across Europe and Canada. GENFI recruited participants with genetic mutations of FTD and their relatives.^6^, ^7^ Participants included carriers of genetic mutations in *C9orf72*, *GRN*, and *MAPT* who have or have not shown symptoms, and their relatives without genetic mutations. Most participants are unaware of their genetic status at recruitment, and remain unaware of their genetic status by a genetic‐guardianship process. Participants underwent a standardized clinical assessment consisting of a medical history, family history, and physical examination. Symptomatic status was based on the assessment by clinicians to determine whether the participants fulfilled the diagnostic criteria for FTD.^11^, ^12^, ^13^ Participants were assessed by the global CDR Dementia Staging Instrument plus National Alzheimer's Coordinating Centre behavior and language domains (CDR plus NACC FTLD),^14^ the Cambridge Behavioural Inventory Revised version (CBI‐R),^15^ and the Mini‐Mental State Examination (MMSE). Participants not diagnosed with FTD who had functional, cerebrovascular, and structural neuroimaging data with good quality were included in this study (*N* = 395). There were 207 FTD genetic mutation carriers who had not shown symptoms fulfilling the diagnostic criteria for FTD at the time of recruitment, termed pre‐symptomatic genetic mutation carriers. There were 188 relatives of the mutation carriers who are not genetic mutation carriers of known FTD genes, termed non‐carriers. The majority of participants scored 0 in their CDR plus NACC FTLD, while 29 pre‐symptomatic genetic mutation carriers and 25 non‐carriers scored 0.5 in their CDR plus NACC FTLD. The demographics and assessment scores of the participants are shown in Table 1. These variables were compared between pre‐symptomatic mutation carriers and non‐carriers using one‐way analysis of variance (ANOVA) for continuous variables and using the chi‐squared test for categorical variables.

**TABLE 1:** Characteristics of participants.

Parameter	Non‐carriers	Pre‐symptomatic mutation carriers	*p*‐value (chi‐squared or ANOVA)
***n***	188	207	
**Age** (years)			
Mean ± SD	45.6 ± 12.1	44.1 ± 11.6	0.23
**Gender**, *n*(%)			
Females	117 (62.2)	139 (67.1)	0.29
Males	71 (37.8)	68 (32.9)	
**Gene**, *n*(%)			
C9orf72	62 (33.0)	76 (36.7)	0.14
GRN	83 (44.1)	97 (46.9)	
MAPT	43 (22.9)	34 (16.4)	
**Mini‐Mental State Examination** Mean ± SD	29.4 ± 1.0	29.4 ± 1.0	0.49
**Cambridge Behavioral Inventory**, mean ± SD	4.6 ± 7.0	6.1 ± 9.7	0.10
**CDR plus NACC FTLD Global Score,** mean ± SD	0.067 ± 0.17	0.070 ± 0.17	0.98

Abbreviation: ANOVA, analysis of variance; CDR plus NACC FTLD, CDR Dementia Staging Instrument plus National Alzheimer's Coordinating Centre behavior and language domains.

### 2.2 Neurocognitive assessment

Participants underwent a neuropsychological battery consisting of tests from the Uniform Data Set,^16^ covering attention and processing speed: Wechsler Memory Scale‐Revised (WMS‐R) digit span forward,^16^ Trail‐Making Test part A (TMTA),^17^ the Wechsler Adult Intelligence Scale‐Revised (WAIS‐R) Digit Symbol Substitution test,^16^ Delis‐Kaplan Executive Function System (DKEFS) Color‐Word Interference Test color and word naming^18^; executive function: WMS‐R Digit span backward,^16^ TMT part B (TMTB),^17^ DKEFS Color‐Word Interference Test ink naming^18^; language: modified Camel and Cactus Test,^19^ the Boston Naming Test (short 30‐item version),^16^ verbal fluency: category fluency and phonemic fluency^16^, ^20^; memory encoding: Free and Cued Selective Reminding Test (FCSRT) immediate free and total recall^21^; memory recall: FCSRT delayed free and total recall, Benson Complex Figure recall^21^; and visuoconstruction: Benson Complex Figure copy. More details of the neurocognitive assessment in this cohort can also be found in the previously published protocol.^6^ A principal component analysis (PCA) with permutation (*n* = 1000) was performed on the series of cognitive measures. Leading components were selected for further investigation.

### 2.3 Neuroimaging acquisition and processing

#### 2.3.1 Gray matter volume

T1‐weighted MRI scans were collected on 3T scanners. A three‐dimensional T1‐weighted magnetization prepared rapid gradient echo sequence image was acquired for each subject accommodating different scanners at each site over at least 283 s (283 to 462 s) and had a median isotropic resolution of 1.1 mm (1 to 1.3 mm), repetition time (TR) of 2000 ms (6.6 to 2400), echo time (TE) of 2.9 ms (2.6 to 3.5 ms), inversion time of 8 ms (8 to 9 ms), and field of view (FOV) 256 × 256 × 208 mm (192 to 256 × 192 to 256 × 192 to 208 mm). For participants with baseline and follow‐up scans, the latest available scans were examined. The co‐registered T1 images were segmented to extract probabilistic maps of six tissue classes: gray matter, white matter, cerebrospinal fluid, bone, soft tissue, and residual noise. The native‐space gray matter and white matter images were submitted to diffeomorphic registration to create equally represented gene‐group template images.^22^ The templates for all tissue types were normalized to the Montreal Neurological Institute (MNI) template using a 12‐parameter affine transformation. The normalized and modulated gray matter volume (GMV) images were used in the analysis.

#### 2.3.2 CBF

ASL sequences could be different across different sites. The sequences included in this study were: pseudo‐continuous ASL 3D fast‐spin‐echo stack‐of‐spirals implemented on a 3T General Electric MR750; pseudo‐continuous ASL 2D gradient‐echo echo‐planar imaging on a 3T Philips Achieva, with and without background suppression; and pulsed ASL 3D gradient‐and‐spin‐echo on 3T Siemens Trio systems. The complete ASL parameters of each sequence have been described elsewhere.^23^

For ASL processing, the ExploreASL pipeline (v1.5.1) was used.^24^ The ExploreASL is optimized for multi‐center data through the use of advanced ASL markers (e.g., spatial coefficient‐of‐variation^25^ and partial volume correction^26^). It has been employed so far in over 30 studies, consisting of ASL scans from three MRI vendors including GE, Philips, and Siemens.^24^ A recent study using this ASL processing method to analyze cerebral perfusion data from the GENFI study has also confirmed the reliability of this method for integrating ASL data from different scanners specific to the GENFI cohort data.^27^ This denoising for scanner effects was complemented with data‐driven and model‐driven correction at the subject level.^28^, ^29^ In this study, structural and functional image volumes across multiple sites, vendors, and sequences were processed first. Briefly, structural images were non‐linearly registered to MNI space using Geodesic Shooting^30^ and transformation matrices were saved for subsequent application on functional images. ASL scans were corrected for motion outliers using rigid‐body transformation coupled with the enhancement of automated blood flow estimates outlier exclusion algorithm,^31^ followed by pairwise subtraction to produce perfusion‐weighted images. Functional proton‐density weighted images were smoothed with a 16 mm full width at half maximum (FWHM) Gaussian kernel to create a bias field that avoided division artifacts during CBF quantification and cancelled out acquisition‐specific B1‐field inhomogeneities. CBF quantification itself followed a single‐compartment model approach and recommendations outlined in the ASL consensus paper.^32^ For quality control, CBF images were reviewed independently by three authors with 3–6 years of experience in handling ASL data. Disagreements were resolved by consensus. CBF volumes were masked by their structural T1 counterpart's probability gray matter mask at ≥50%, and the spatial coefficient of variation was calculated for the extracted voxels. Images with a coefficient of variation values ≥0.8 were discarded.

To adjust for differences arising from the effects of multiple sites, scanners, and software, a spatially varying intensity normalization approach was used,^8^ together with data‐driven and model‐driven approaches at the between‐subject level (see section Statistical analysis). The normalization approach uses the within‐site CBF similarity between participants to remove the between‐site quantification differences.^8^ Mean CBF images of these groupings were calculated and smoothed using a 6.4 mm FWHM Gaussian kernel. Smoothing was constrained to a binary MNI brain mask. These group‐specific mean images were then averaged to calculate the population mean CBF image, which in turn was rescaled uniformly such that the mean gray matter perfusion equaled 60 mL/min/100 g. Group‐specific rescale‐factor images were then calculated by dividing this population CBF image by each group's mean CBF image. Individual CBF images were adjusted via multiplication against their group's respective rescale‐factor image. To account for the effects of atrophy, partial volume correction on rescaled CBF volumes was performed using a linear regression approach.^26^ Further details of ASL processing are discussed in a recent publication.^27^ Due to hyperintensities present in the cerebellum of many subjects which is not our interest of study, only the CBF of the cortical region was included in the analysis of this study. A cortical binary mask created from the Harvard‐Oxford cortical atlas (https://fsl.fmrib.ox.ac.uk/fsl/fslwiki/Atlases) was therefore applied to all CBF images.

#### 2.3.3 Resting‐state functional networks

For rs‐fMRI, echo planar imaging acquired 200 volumes with 42 slices (slice thickness = 3.5 mm, TR = 2500 ms; TE = 30 ms; FOV = 192 mm × 192 mm). Resting‐state fMRI data were preprocessed using Automatic Analysis^33^ calling functions from SPM12 implemented in Matlab (MathWorks). Processing steps included (1) spatial realignment to correct for head movement and movement by distortion interactions, (2) temporal realignment of all slices, and (3) coregistration of the echo planar imaging to the participant's T1 anatomical scan. The normalization parameters from the T1 stream were applied to warp functional images into MNI space. Resting‐state fMRI data were further processed using whole‐brain independent component analysis (ICA) of single‐subject time series denoising, with noise components selected and removed automatically using the ICA‐based Automatic Removal of Motion Artifacts toolbox.^34^ This was complemented with linear detrending of the fMRI signal, covarying out six realignment parameters, white matter and cerebrospinal fluid signals, their first derivatives, and quadratic terms.^35^ Global white matter and cerebrospinal fluid signals were estimated for each volume from the mean value of white matter and cerebrospinal fluid masks derived by thresholding SPM tissue probability maps at 0.75. Data were band‐pass filtered using a discrete cosine transform.

To identify the activation of functional networks from rs‐fMRI, group‐level ICA was performed to decompose the rs‐fMRI data^36^ from 395 participants (including pre‐symptomatic mutation carriers and non‐carriers). ICA dissociates signals from complex datasets with minimal assumptions, to represent data in a small number of independent components (ICs) which here are spatial maps that describe the temporal and spatial characteristics of underlying signals.^36^, ^37^ The values at each voxel reflect the correlation between the time series of the voxel and that of the component. Each component can, therefore, be interpreted as blood oxygen level dependent (BOLD) co‐activation across voxels of a functional network at a resting state. The number of components used, *N* = 15, matched a common degree of decomposition previously applied in low‐dimensional group‐ICA of rs‐fMRI^38^, ^39^, ^40^ and generated network spatial maps that showed a high degree of overlapping with network templates. Low‐dimensional group‐ICA was used because the purpose was to define each network with a single component, and high‐dimensional group‐ICA would tend to decompose a single network into multiple components. The stability of the estimated ICs was evaluated across 100 ICASSO iterations.^41^ Functional networks were identified from components by visualization and validated by spatially matching the components to pre‐existing templates,^42^ in accordance with the previous methodology used to identify networks from ICs.^43^ The dorsal and ventral default mode network, the salience network, and the left and right frontoparietal network were selected, which are higher‐order functional networks known to be associated with age‐ and FTD‐related cognitive change.^44^, ^45^, ^46^

### 2.4 Statistical analysis

#### 2.4.1 Linked ICA

**FIGURE 1:**
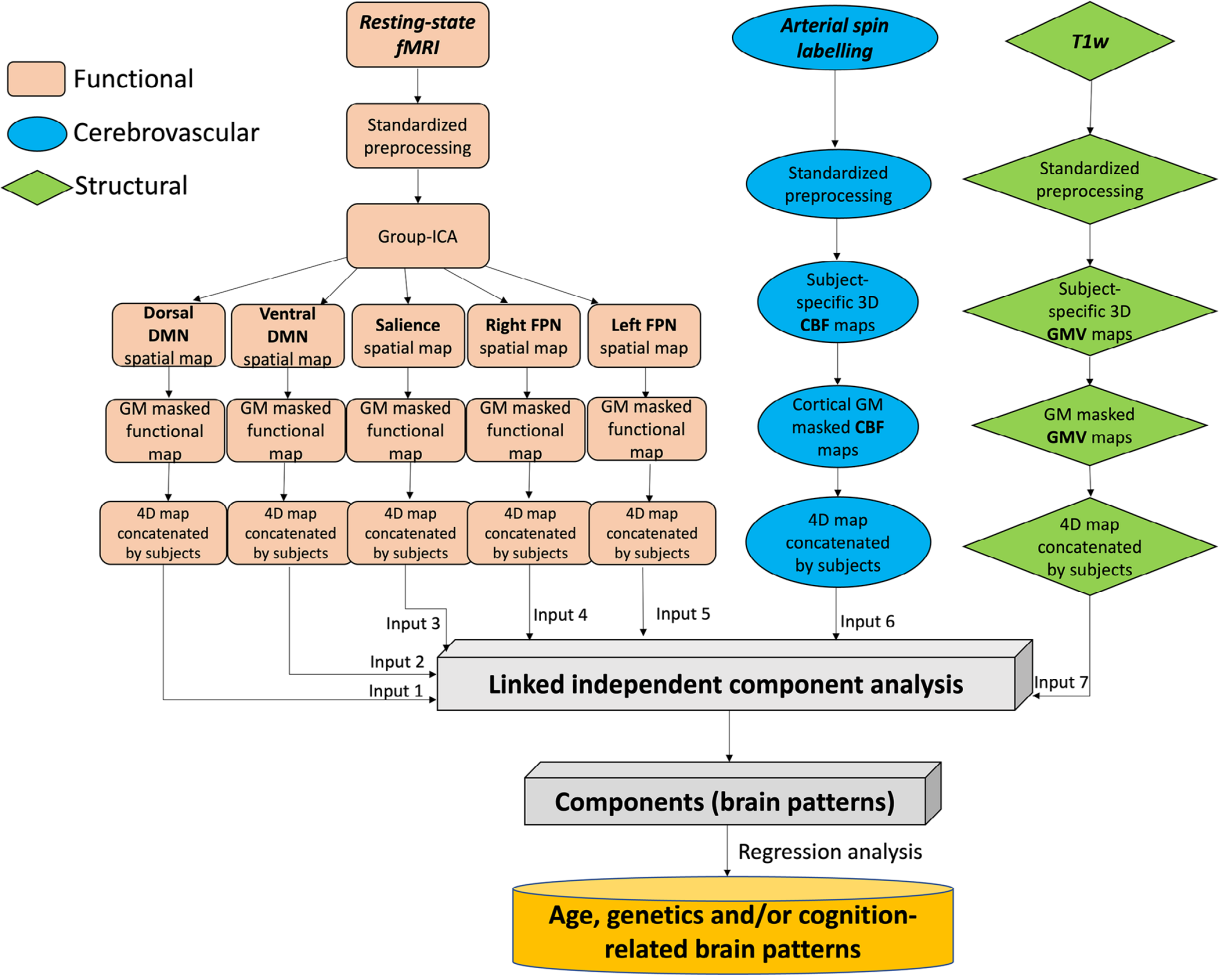
Summary of processing and analysis of the imaging modalities, comprising functional, cerebrovascular, and structural measurements. CBF, cerebral blood flow; DMN, default mode network; FPN, frontoparietal network; GMV, gray matter volume; ICA, independent component analysis; fMRI, functional magnetic resonance imaging; SN, salience network; T1w, T1‐weighted.

Linked independent component analysis (ICA) was performed using FLICA of FMRIB^47^, ^48^ implemented in Matlab (MathWorks version 2021b). Linked ICA is a data‐driven analytic method that allows for the simultaneous characterization of multimodal imaging modalities while taking into account the covariance across imaging modalities.^47^ In comparison with other commonly used multivariate approaches for multivariate data integration such as canonical correlation analysis and partial least squares, linked ICA is able to identify patterns of covariance across more than two modalities. Linked ICA was run with seven spatial map inputs: GMV, CBF, and five co‐activation maps from resting‐state functional networks (i.e., the dorsal default mode network, the ventral default mode network, the salience network, the right frontoparietal network, and the left frontoparietal network) identified as described in 2.3.3. To ensure the results were not influenced dominantly by non‐gray matter regions (e.g., ventricles), all spatial maps were masked by thresholding SPM gray matter tissue probability maps at 0.3. We refer to these imaging‐derived spatial maps as modalities in linked ICA. A summary flow chart of the processing and analysis of imaging modalities is presented in Figure 1.

Within each modality, images from all subjects were concatenated into a single input matrix (participants‐by‐voxels) for linked ICA. Linked ICA decomposed this n‐by‐m matrix of participants‐by‐voxels into spatial components, with each component being an aggregate of spatial patterns, one for each modality, along with a set of subject loadings, one for each component.^47^ Each modality spatial pattern is a map of weights that is later converted to a pseudo‐Z‐statistic by accounting for the scaling of the variables and the signal‐to‐noise ratio in that modality. Only modalities with significant contribution (i.e., weighting with Z‐score > 3.34, which corresponds to *p* < 0.001) were presented in this study. Linked ICA subject loadings for a given component were shared among all modalities represented in that component and indicated the degree to which that component was expressed by any individual subject. Subject loadings were used as inputs to the second‐level between‐subject regression analysis (see below in 2.4.2).

#### 2.4.2 Multiple regression analysis

To investigate the effects of age (linear and quadratic) and genetic mutation on cognition, multiple regression analysis was used with cognition PCA component scores as the dependent variable. The group was classified by genetic mutation status (i.e., pre‐symptomatic mutation carriers or non‐carriers). Gender and site effect were included as covariates. In Wilkinson's notation,^49^ the model took the form:

$$\mathrm{Cognition}\;\mathrm{component}∼\mathrm{Group}*{\mathrm{Age}}^{2}+\mathrm{Gender}+\mathrm{Site}.$$

To investigate whether brain patterns were predicted by age (linear and quadratic), genetic mutation, and their interaction, subject loadings of each linked ICA component (IC) of interest were investigated as the dependent variable in multiple regression. Gender, total brain volume, and site effect were included as covariates. In Wilkinson's notation, the model took the form:

$$\mathrm{IC}∼\mathrm{Group}*{\mathrm{Age}}^{2}+\mathrm{Gender}+\mathrm{Total}\;\mathrm{brain}\;\mathrm{volume}+\mathrm{Site}.$$

Finally, to investigate the relationship between brain patterns and cognitive variability, accounting for the effects of genetics and age (linear and quadratic), multiple regression was used taking the following form:

$$\begin{array}{ccc} & & \mathrm{Cogn}\mathrm{ition}\;\mathrm{comp}\mathrm{onent}∼\mathrm{IC}*\mathrm{Group}*\mathrm{Ag}e^{2}+\mathrm{Gender}\\ & & \;+\;\mathrm{Total}\;\mathrm{brain}\;\mathrm{volume}+\mathrm{Site}. \end{array}$$

A false discovery rate (FDR)‐corrected *p* < 0.05 was considered statistically significant. Analyses were performed in Matlab.

## 3 RESULTS

### 3.1 Relationship between age, gene group, and cognitive function

**FIGURE 2:**
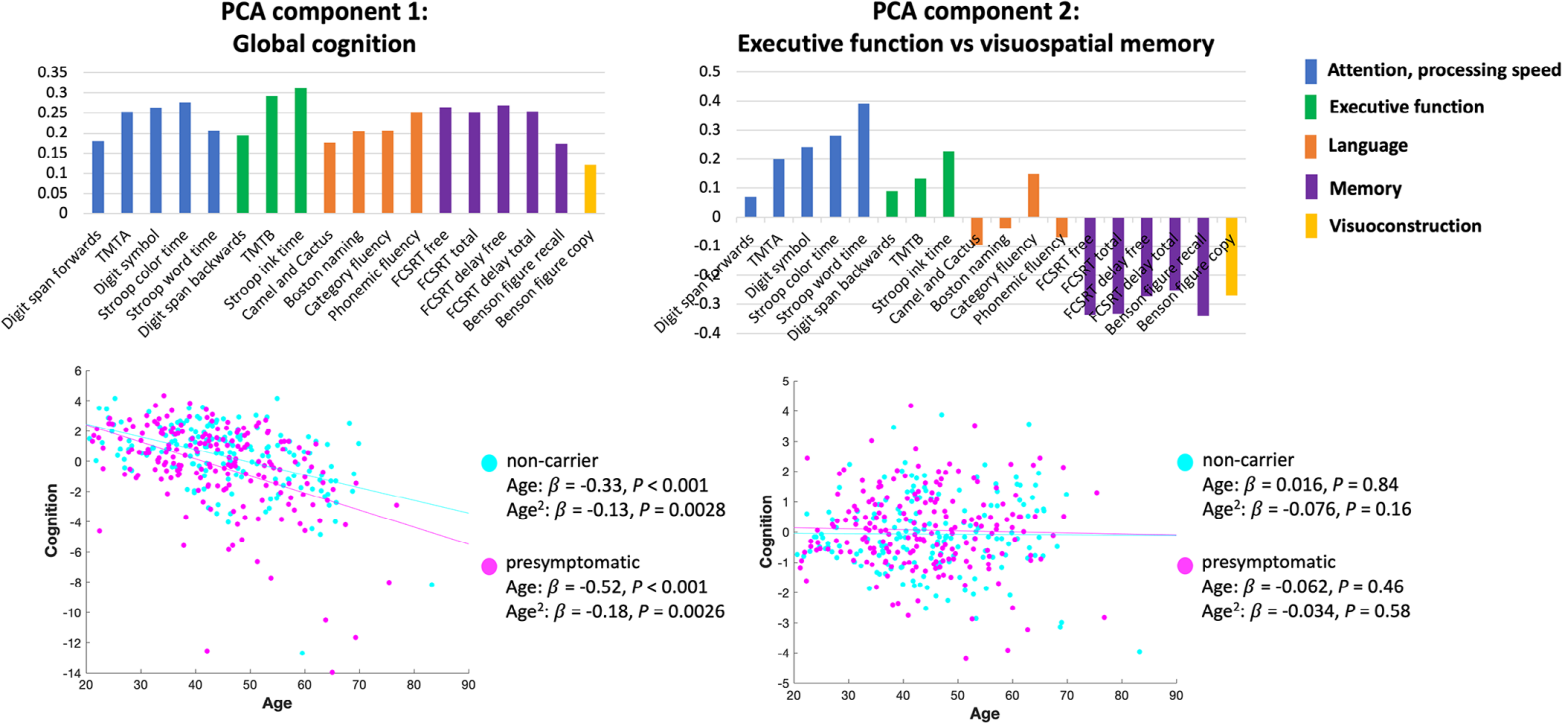
The two significant components from principal component analysis (PCA) on cognitive assessments. The top row shows the loadings of each cognitive test in PCA components. The bottom row shows the scatter plots of the correlation between age and PCA cognition component scores.

The two significant PCA components are shown in Figure 2. The first cognition component (variance explained 36.6%, *p* < 0.001) was related to global cognitive function. No significant group‐wise difference in global cognition was found between genetic mutation carriers and non‐carriers (*p* = 0.079). Both non‐carriers and pre‐symptomatic genetic mutation carriers showed a decline in global cognition with age likely reflecting the general age‐related decrease in global cognitive function. No significant difference was found in the age‐cognition relationship between genetic mutation carriers and non‐carriers (Group:Age interaction *t* = −0.97, *p* = 0.33; Group:Ageˆ2 interaction *t* = −0.73, *p* = 0.47).

The second cognition component (variance explained 9.1%, *p* < 0.001) indicated executive function, attention, and processing speed with deficits in visuospatial memory. No significant group‐wise difference was found between genetic mutation carriers and non‐carriers (*p* = 0.28). Neither non‐carriers nor pre‐symptomatic genetic mutation carriers showed age‐related changes in these cognitive functions. No significant difference was found in the age‐cognition relationship between genetic mutation carriers and non‐carriers (Group:Age interaction *t* = −0.62, *p* = 0.53; Group:Ageˆ2 interaction *t* = 0.58, *p* = 0.56).

### 3.2 Multimodal fusion using linked ICA

**FIGURE 3:**
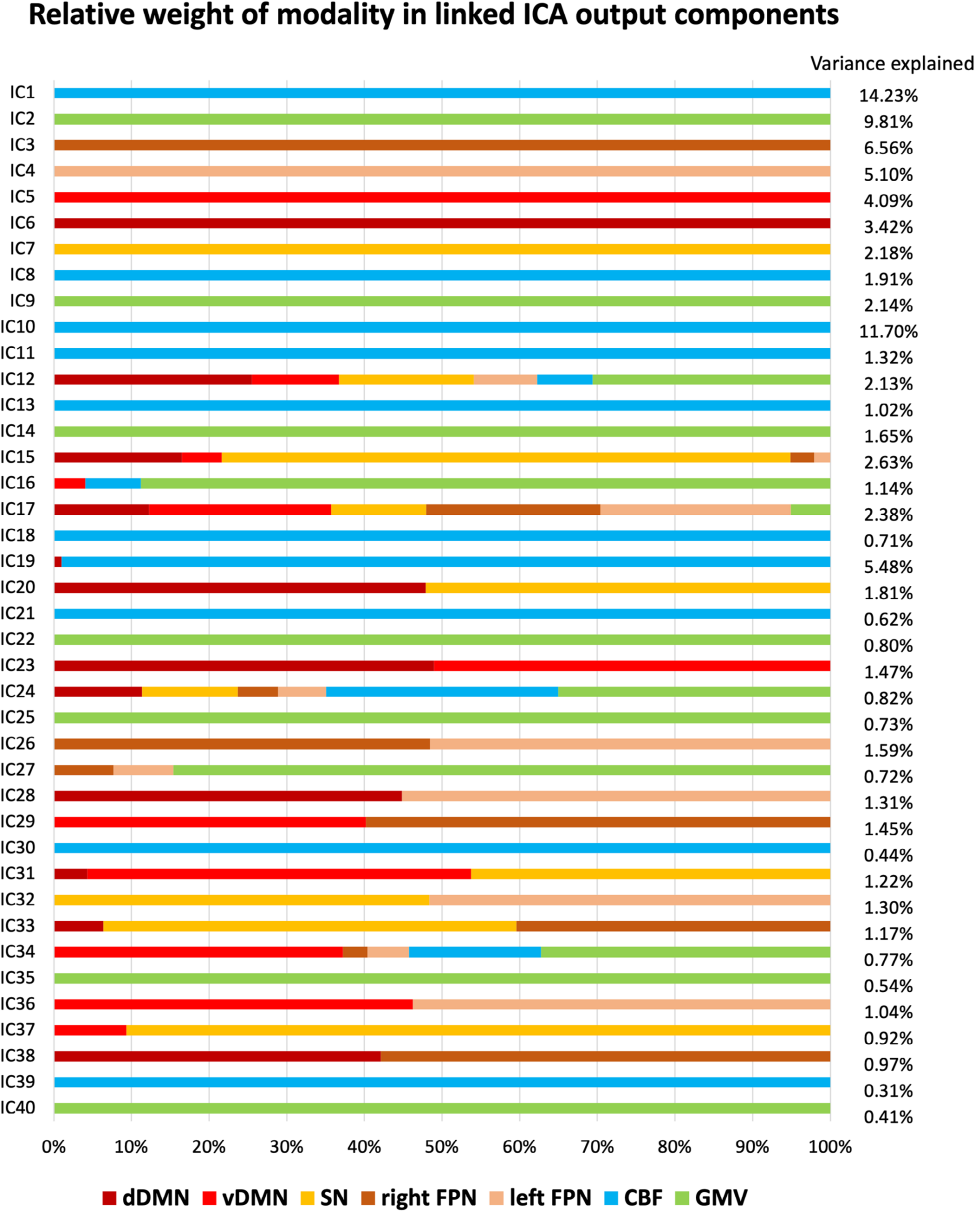
The relative weight of modalities in each component generated from linked independent component analysis (ICA) and the percentage of variance explained of each component. CBF, cerebral blood flow; dDMN, dorsal default mode network; FPN, frontoparietal network; GMV, gray matter volume; SN, salience network; vDMN, ventral default mode network.

The relative weight of modalities in each linked ICA output component is shown in Figure 3. Three components (IC10, IC14, and IC19) were excluded from further analysis as they were dominated by signals from one or two subjects (e.g., due to regional hyperintensities reflected by ASL images). We focused on components with variance explained >1%. Note that there was little fusion between functional signals and structural or vascular signals.

### 3.3 Relationship between age, gene group, and neuroimaging components

**FIGURE 4:**
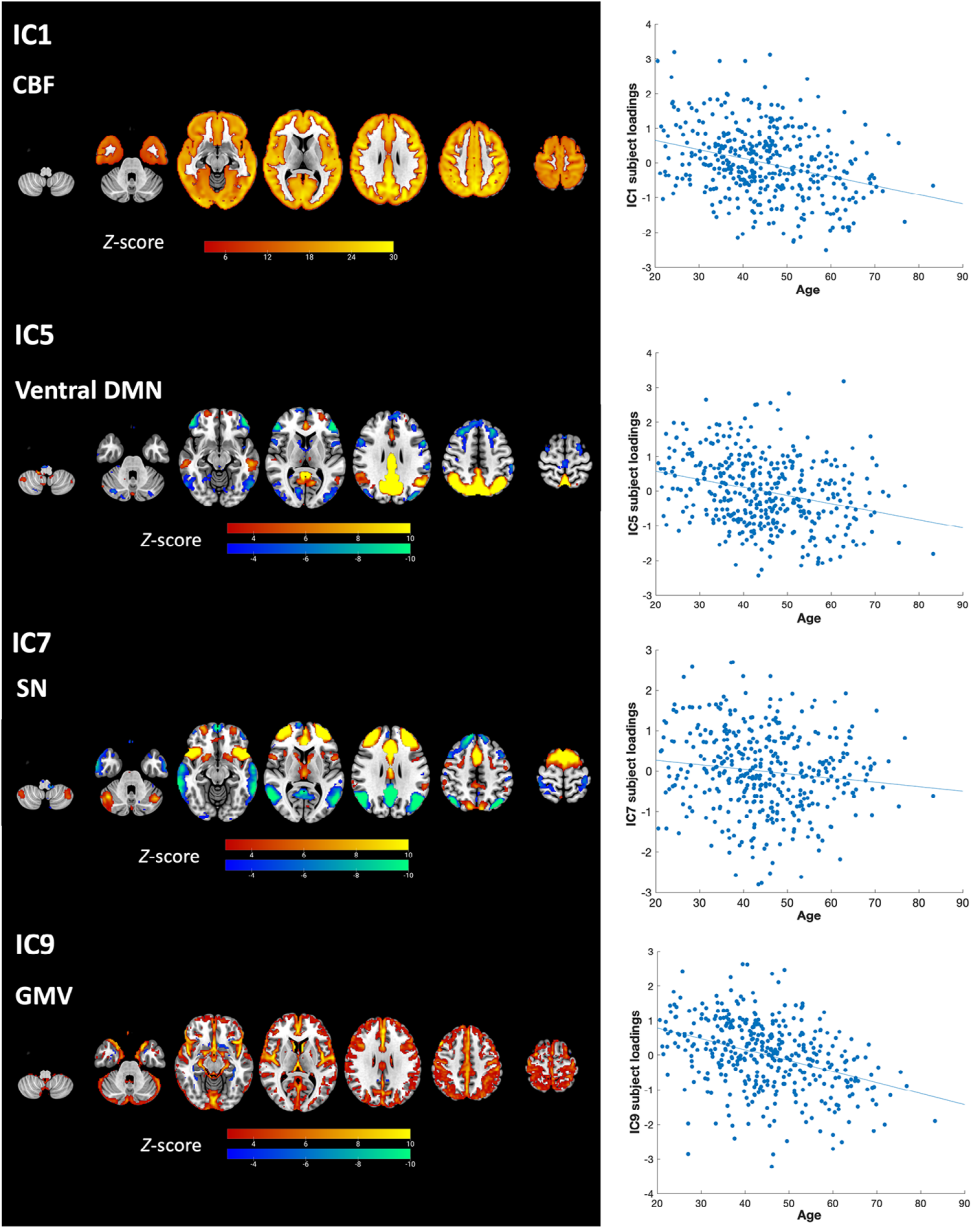
Brain visualization and scatter plots of subject loadings against age of the linked independent component analysis components (ICs) showing strong age effects.

**FIGURE 5:**
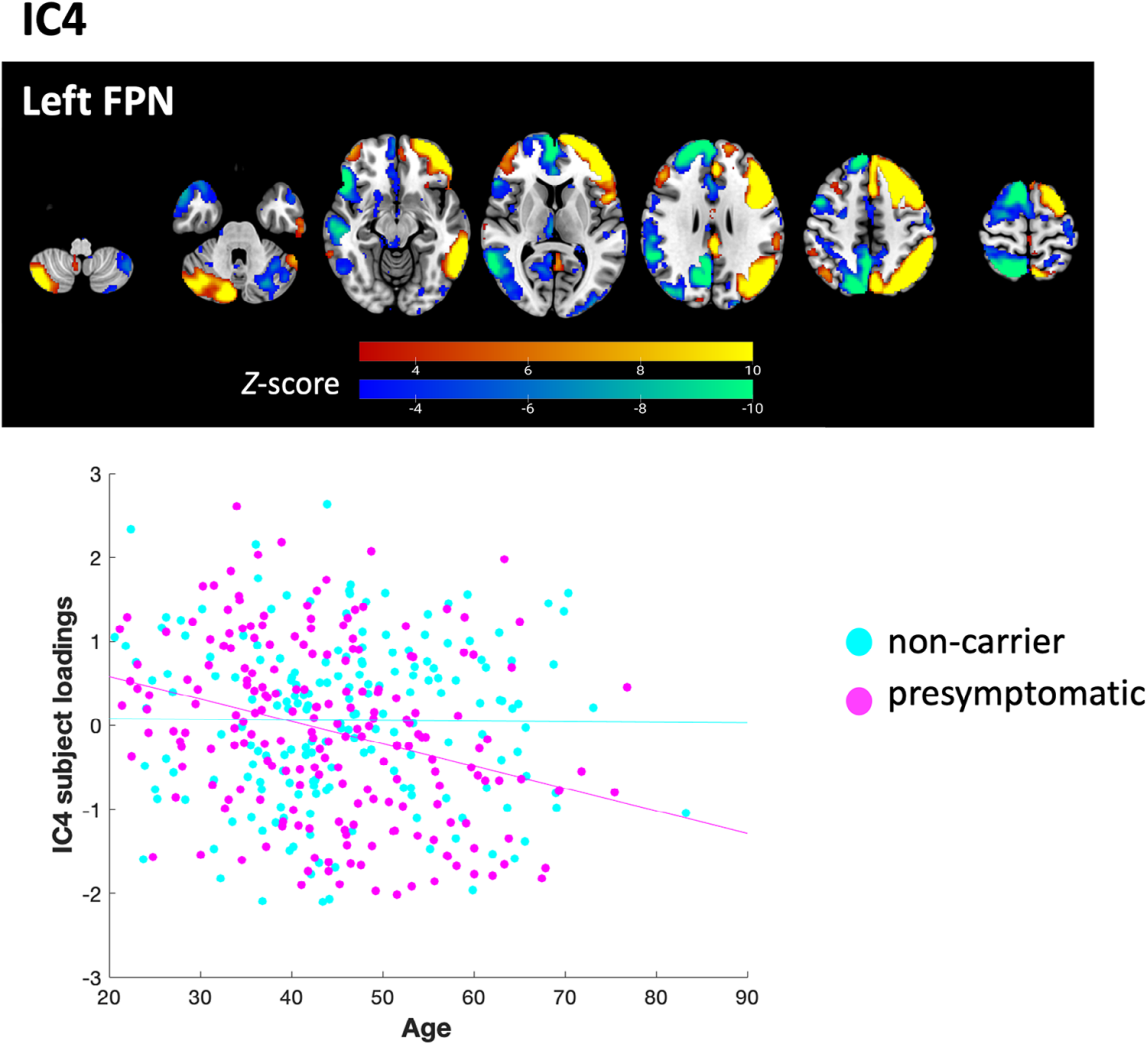
Brain visualization of linked independent component analysis component 4 (IC4), showing the left frontoparietal network (FPN). For visualization, the brain spatial map threshold is set to 3 < |Z| < 10. The scatter plot shows the correlation between age and IC4 subject loadings, separated by pre‐symptomatic genetic mutation carriers (*r* = −0.30, *p* < 0.001) and non‐carriers (*r* = −0.0087, *p* = 0.91).

Multiple regression analysis results of the linked ICA components of interest are shown in Table 2. We focused on components with a significant model fit (FDR‐corrected *p* < 0.05 for adjusted *R* ^2^, i.e., the components that showed significant correlations with the variables being tested). Strong linear age effects were observed particularly in components indicating global CBF (IC1), ventral default mode network (IC5), salience network (IC7), and head motion (IC9) (Figure 4). Only one component, IC4, showed differential age effects between pre‐symptomatic and non‐carriers (Group:Age interaction *t* = −2.82, *p* = 0.0051). As age increased, pre‐symptomatic genetic mutation carriers showed decreased activation of the left frontoparietal network (IC4, *r* = −0.30, *p* < 0.001), while non‐carriers did not (*r* = −0.0087, *p* = 0.91). Brain visualization of IC4 and its scatter plot against age are shown in Figure 5. Further analyses to examine for possible specificity to *GRN*, *MAPT*, or *C9orf72* carriers showed that the interaction between genetic mutation status and age (Group:Age) in the regression model was significant within the *GRN* mutation carriers (Group:Age interaction *t* = −2.44, *p* = 0.016), but was not significant in the rest of the pre‐symptomatic genetic mutation carriers excluding *GRN* mutation carriers (Group:Age interaction *t* = −1.43, *p* = 0.16). It was neither significant within the *C9orf72* mutation carriers (Group:Age interaction *t* = −1.53, *p* = 0.13) nor within the *MAPT* mutation carriers (Group:Age interaction *t* = −1.42, *p* = 0.16) alone. Brain spatial maps of other components are presented in Figure S1.

**TABLE 2:** Multiple regression results of the linked independent component analysis components of interest (IC).

IC ∼ Group*Age^2^ + Gender + Total brain volume + Site															
		Model		Group		Gender		Age		Age^2^		Group:Age		Group:Age^2^	
IC	Variance explained	Adjusted *R* ^2^	*p*	*β*	*p*	*β*	*p*	*β*	*p*	*β*	*p*	*β*	*p*	*β*	*p*
IC1	14.23%	0.14	**<0.001**	−0.17	**0.0048**	−0.26	**<0.001**	−0.36	**<0.001**	0.012	0.74	−0.011	0.82	−0.0032	0.93
IC2	9.81%	0.59	**<0.001**	0.049	0.25	0.27	**<0.001**	−0.082	**0.031**	−0.0085	0.74	0.036	0.29	−0.0049	0.85
IC3	6.56%	0.34	**<0.001**	−0.080	0.14	0.090	0.075	−0.11	**0.022**	−0.029	0.38	−0.029	0.51	0.041	0.22
IC4	5.10%	0.27	**<0.001**	−0.078	0.17	0.067	0.21	−0.13	**0.012**	0.032	0.36	−0.13	**0.0051**	0.034	0.33
IC5	4.09%	0.16	**<0.001**	0.060	0.33	−0.052	0.36	−0.29	**<0.001**	0.038	0.31	−0.092	0.062	−0.013	0.73
IC6	3.42%	0.12	**<0.001**	−0.067	0.28	−0.16	**0.0067**	−0.15	**0.0058**	0.038	0.31	−0.084	0.10	0.068	0.077
IC7	2.18%	0.20	**<0.001**	0.012	0.84	−0.024	0.66	−0.20	**<0.001**	0.075	**0.040**	−0.085	0.075	0.028	0.44
IC8	1.91%	0.050	**0.013**	0.11	0.10	0.26	**<0.001**	0.050	0.38	0.054	0.17	−0.039	0.45	−0.035	0.38
IC9	2.14%	0.47	**<0.001**	−0.068	0.16	−0.62	**<0.001**	−0.30	**<0.001**	−0.049	0.10	0.025	0.53	0.032	0.28
IC12	2.13%	0.79	**<0.001**	0.040	0.18	0.12	**<0.001**	−0.017	0.53	−0.024	0.20	−0.0031	0.90	−0.026	0.17
IC13	1.02%	0.038	**0.039**	−0.036	0.58	−0.0081	0.89	0.072	0.22	−0.11	**0.0061**	−0.0035	0.95	0.022	0.58
IC15	2.63%	0.10	**<0.001**	0.039	0.54	−0.12	**0.047**	0.0087	0.88	0.042	0.27	−0.048	0.35	0.0059	0.88
IC16	1.14%	0.28	**<0.001**	0.031	0.59	−0.22	**<0.001**	0.24	**<0.001**	0.074	**0.032**	0.073	0.11	0.022	0.53
IC17	2.38%	0.33	**<0.001**	−0.013	0.81	−0.12	**0.018**	−0.082	0.093	−0.0083	0.80	0.0092	0.84	−0.016	0.63
IC20	1.81%	0.063	**0.0032**	0.013	0.85	0.070	0.24	−0.10	0.10	0.029	0.46	−0.0016	0.98	−0.018	0.65
IC23	1.47%	0.053	**0.0096**	0.050	0.44	−0.050	0.41	0.053	0.36	−0.0083	0.83	−0.088	0.093	−0.030	0.45
IC26	1.59%	0.037	**0.043**	−0.012	0.85	0.22	**<0.001**	0.075	0.20	−0.0034	0.93	−0.039	0.46	−0.015	0.70
IC29	1.45%	0.066	**0.0024**	0.025	0.70	0.046	0.44	0.12	**0.033**	0.066	0.10	0.017	0.74	0.042	0.28
IC31	1.22%	0.067	**0.0022**	0.0084	0.90	−0.10	0.10	−0.065	0.26	−0.067	0.090	−0.088	0.093	−0.00040	0.99
IC33	1.17%	0.039	**0.036**	−0.082	0.21	−0.021	0.73	0.0039	0.95	−0.052	0.20	−0.033	0.54	0.032	0.42

*Note*: *p*‐values in bold are statistically significant.

### 3.4 Relationship between neuroimaging components and cognitive function

All linked ICA components that showed cognition‐related differences between the two groups reflected a single neuroimaging modality. No component showed a different association with cognition component 1 between non‐carriers and pre‐symptomatic mutation carriers (Table S1).

**FIGURE 6:**
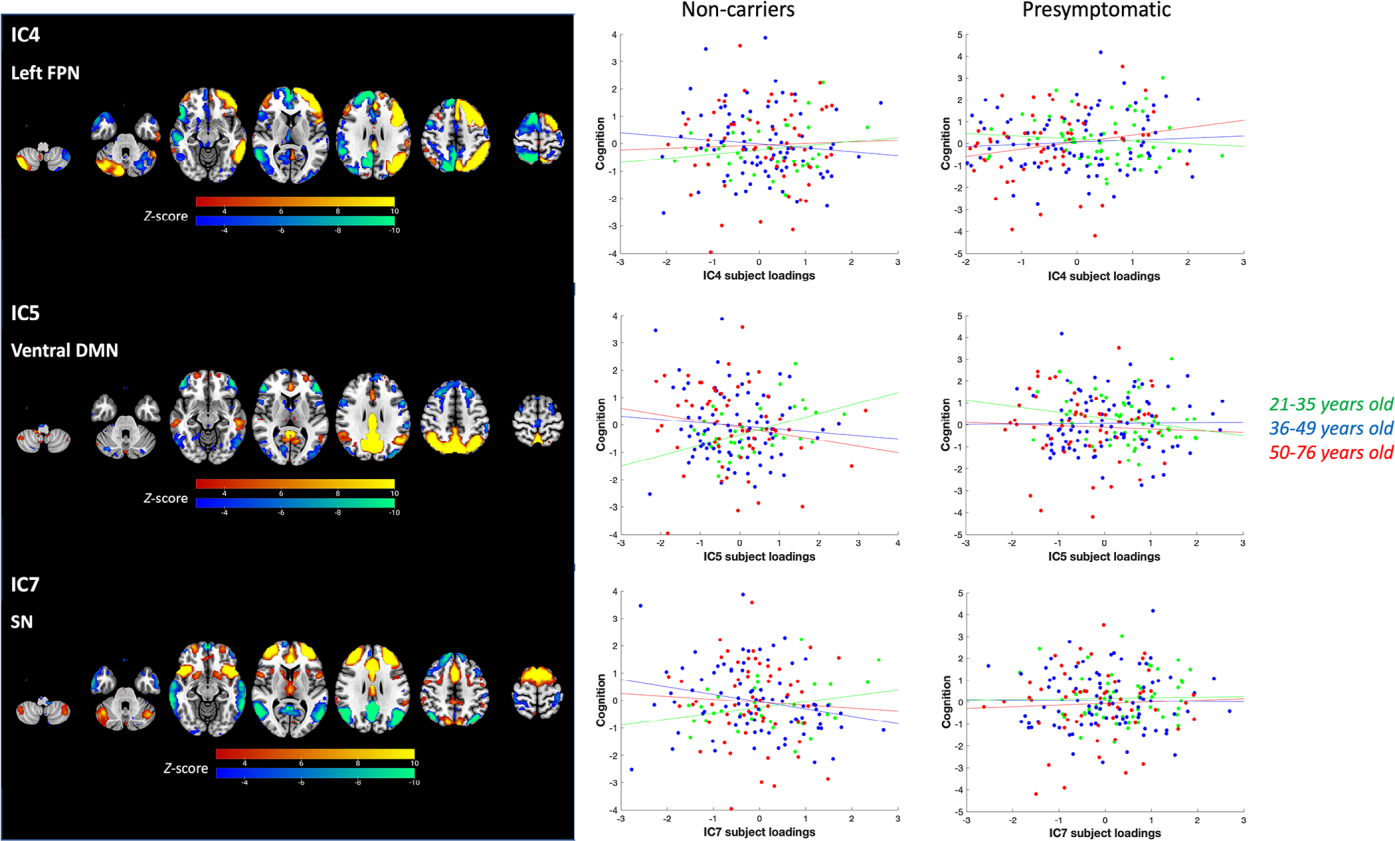
Linked independent component analysis components showing three‐way interactions between subject loadings with group (genetic mutation) and age in predicting cognition component 2. IC4 indicates the left frontoparietal network (FPN), IC5 indicates the ventral default mode network (DMN), and IC7 indicates the salience network (SN). The brain visualization and scatter plots are shown. The scatter plots show the correlation between linked independent component analysis (IC) subject loading scores and principal component analysis (PCA) cognition component 2 scores, for visualization purpose separated by pre‐symptomatic genetic mutation carriers and non‐carriers and three age groups.

In regards to component 2 (Table S2), IC2, indicating global GMV, showed an interaction with genetic mutation in predicting cognition component 2 (IC:Group *t* = −2.73, *p* = 0.0066): non‐carriers showed a positive association between IC2 subject loadings and good performance on executive functions and poor performance on visuospatial memory tasks (*r* = 0.17, *p* = 0.026), while this association was not significant in pre‐symptomatic mutation carriers (*r* = −0.12, *p* = 0.10). There was a significant three‐way interaction between group, age, and IC subject loadings of the left frontoparietal network (i.e., IC4, IC:Group:Ageˆ2 *t* = −2.20, *p* = 0.029) in predicting cognition component 2. Visualizing the results (Figure 6) indicates that as age increased, an increased association between the left frontoparietal network and good performance on executive functions and poor performance on visuospatial memory tasks was found in pre‐symptomatic genetic mutation carriers. This result was confirmed in a post‐hoc test showing that a significant two‐way interaction between IC4 and age in predicting these cognitive performances was found in pre‐symptomatic genetic mutation carriers (IC:Ageˆ2 *t* = −2.14, *p* = 0.033) but not in non‐carriers (IC:Ageˆ2 *t* = 1.70, *p* = 0.090). Significant 3‐way interactions (IC:Group:Ageˆ2) were also observed for the component of ventral default mode network (IC5, *t* = −2.73, *p* = 0.0068) and salience network (IC7, *t* = −3.14, *p* = 0.0018). The effects in both components suggested an age‐varying association between network activity and performance on executive functions and visuospatial memory in non‐carriers but not in pre‐symptomatic mutation carriers (Figure 6).

In a post‐hoc analysis to examine the relationship between age and executive functions, which are the most commonly affected cognitive domains in FTD, we have selected only the tests examining executive functions, attention, and processing speed and performed a PCA on them (Figure S2). We examined the relationship between age and the significant PCA component (i.e., principal component (1) representing the overall performance of these tests. Results showed a negative association between age and this component in both pre‐symptomatic mutation carriers (Age *t* = −6.78, *p* < 0.001; Ageˆ2 *t* = −2.73, *p* = 0.007) and non‐carriers (Age *t* = −4.21, *p* < 0.001; Ageˆ2 *t* = −3.37, *p* < 0.001).

## 4 DISCUSSION

In this study, we co‐analyzed GMV, CBF, and functional network integrity. Interplay across modalities did not relate to genetic groups or cognition. Pre‐symptomatic genetic mutation carriers showed a decrease with age in the left frontoparietal network integrity while non‐carriers did not, suggesting a gene‐related neurodegenerative consequence above normal aging. Executive functions of pre‐symptomatic mutation carriers dissociated from the level of atrophy but became dependent on the left frontoparietal network integrity with age. Results suggest that maintaining frontoparietal network integrity may support cognitive function despite age‐related atrophy and hypoperfusion in pre‐symptomatic FTD.

The age‐related decreases in CBF and default mode network activity found in this study are consistent with the commonly observed changes in perfusion^50^, ^51^ and functional network^52^ in normal aging. Global GMV also decreased with age, consistent with previous multimodal neuroimaging fusion studies^43^, ^53^ and aging pattern of the brain.^54^ The component representing global GMV (IC2) in this study did not significantly differ between pre‐symptomatic mutation carriers and non‐carriers accounting for age. The main reason may be this component is dominated by the effect of aging, as linked ICA identifies components in a data‐driven manner. Signals in this component are mostly influenced by age‐related variances, which can be attributed to the wide age range of participants, spanning from 20 to 83 years old. On the other hand, studies employing hypothesis‐driven approaches identified atrophy patterns that are optimized to detect pre‐symptomatic differences.^4^, ^7^ Thus, the difference in atrophy patterns identified in those studies might be specific to pre‐symptomatic mutation carriers versus age‐matched controls,^7^, ^55^ while IC2 in our study predominantly reflects age‐related atrophy as reported in previous studies.^43^, ^53^, ^56^

More importantly, we illustrated the age‐ and cognition‐relevant divergence of frontoparietal network integrity between pre‐symptomatic mutation carriers and non‐carriers. Pre‐symptomatic mutation carriers showed a decrease in left frontoparietal network integrity with age, while non‐carriers did not, suggesting that the lateralized frontoparietal network is the functional network most affected by FTD mutations with age. Salience network connectivity is commonly reduced in symptomatic behavioral variant FTD (bvFTD) and associated with disease severity,^45^, ^57^ but remains unchanged at the pre‐symptomatic stage.^58^ Altered default mode network connectivity has been found in both pre‐symptomatic *MAPT* mutation carriers and bvFTD subjects.^45^, ^58^ In this study, we did not find the default mode network or the salience network significantly different between genetic mutation carriers and non‐carriers. Nevertheless, when relating to executive function, attention, and processing speed, the associations of the ventral default mode network and the salience network, respectively, with performance in these functions were found in younger non‐carriers but not in pre‐symptomatic mutation carriers, suggesting cognitive reliance on these functional networks breaks down in genetic mutation carriers and during aging. Understanding such an effect would be important for gaining insights into the mechanisms of cognitive decline and the maintenance of executive functions.

Although pre‐symptomatic mutation carriers maintain similar global cognition to non‐carriers, they showed a trend of more rapid decline in global cognition with age. We found no significant association between age and executive function, attention, and processing speed in either group, contrasting previous reports of age‐related declines in executive functions,^59^ potentially due to different analysis methods. Here, executive functions were represented by the second principal component, which should be interpreted in relation to the negatively loading visuospatial memory and in the context of the first principal component. Principal component 1 captured the well‐documented age‐related decline in global cognition including executive functions and memory.^60^, ^61^ Principal component 2 may represent aspects of executive functions, that are independent of the general cognitive decline, possibly reflecting individual variability specific to the cognitive tests. Hence, the age‐related differences in these executive functions might be moderated by the age‐related effect of visuospatial memory, while executive functions declining alongside memory are likely already captured by principal component 1. Post‐hoc analysis showed a negative age‐executive functions association, consistent with reported age‐related executive declines.^59^ The performance related to attention, processing speed, and executive function correlated with global GMV in non‐carriers, while correlated with left frontoparietal network integrity in pre‐symptomatic mutation carriers especially as they get older. It suggests that in genetic mutation carriers, executive functions dissociated from GMV and were maintained by frontoparietal network integrity. Frontoparietal network is important for cognitive flexibility especially for executive function,^62^, ^63^ one of the most commonly affected cognitive domains in FTD.^1^ A recent study found that pre‐symptomatic *C9orf72* mutation carriers showed lower attention and executive function compared to non‐carriers.^61^ Our study provides further evidence suggesting that these cognitive domains are sensitive to alternations at the earlier stage of the disease. Given that CBF and GMV significantly decreased with age regardless of genetic mutations, and the reliance on other functional networks for cognitive performance broke down in genetic mutation carriers, maintaining frontoparietal network integrity might be the key to slowing cognitive decline, particularly executive declines, at the pre‐symptomatic stage of FTD.

The atrophy patterns can be different across different genetic mutations. The *GRN* genetic mutation is known for causing asymmetric atrophy while the atrophy patterns of FTD associated with *MAPT* genetic mutation are typically symmetric.^7^, ^55^, ^64^ We observed asymmetric relationship between functional network integrity and age in *GRN* mutation carriers, indicating that the asymmetric vulnerability to genetic mutation can be manifested at the pre‐symptomatic stage. Specifically, we observed a relationship between age and the left frontoparietal network in *GRN* mutation carriers, although the lack of significance in other genetic groups may be attributed to smaller sample sizes compared to *GRN* mutation carriers. Such finding is consistent with previous studies showing selective vulnerability of the left hemisphere.^55^, ^65^, ^66^ Moreover, there is inherent asymmetry in several human cognitive systems, including language and executive functions, which could be significantly impaired in FTD.^67^, ^68^, ^69^ Although the cellular mechanisms of selective vulnerability are not well understood, it would be important to investigate the laterality of changes in future studies, especially considering the dynamical interactions between brain networks which shape cognition.

This study benefits from pathological confidence arising from genetic characterization, and the large sample size of pre‐symptomatic mutation carriers through the multi‐center GENFI study. This study combines GMV, CBF, and functional networks in pre‐symptomatic FTD genetic mutation carriers. Linking neurobiological changes is important given potential synergistic effects. Although, we found no interplay across modalities, relating the frontoparietal network to other unexplored pathologies like tau, amyloid, and neurotransmitters may be informative,^45^, ^58^, ^70^, ^71^ given its age‐ and cognition‐related distinctions between genetic mutation carriers and non‐carriers observed in our study.

The study also has limitations. First, the variability of MRI acquisition scanners and sequences through the multi‐center cohort is higher than in a single‐center study. However, we mitigated the effects through the use of normalization, denoising, and statistical adjustment for side effects. We recognize that multi‐center and multi‐scanner correction for ASL could potentially be improved. A standard approach would be the use of flow phantoms for calibrating a scanner's ASL signal to a ground‐truth flow rate.^72^ Currently, however, this is not implemented in most ASL studies. Existing methods of pre‐model or within‐model corrections^73^ along with data‐driven and model‐driven corrections for sites and scanners remain the most pragmatic approach. Second, this study is cross‐sectional. This should be noted when interpreting age effects, as dynamic aging effects require longitudinal data. More follow‐up visits of the ongoing GENFI cohort will allow a longitudinal examination. Third, only adults were included, thus potentially missing the changes manifested before adulthood caused by genetic mutation. A new cohort within GENFI is starting which aims to study family members below the age of 18. Fourth, there were some pre‐symptomatic genetic mutation carriers with a CDR plus NACC FTLD global score of 0.5, indicating that they might have mild clinical symptoms but were not diagnosed as FTD. However, the pre‐symptomatic mutation carriers did not differ from non‐carriers in their groupwise CDR plus NACC FTLD score, CBI‐R, or MMSE. This suggests that the difference in functional networks observed in this study is not likely to be related to mis‐assigned early‐symptomatic patients carrying mutations. Future studies can implement a more refined and multidimensional classification of the pre‐symptomatic stage, such as the mild cognitive and/or behavioral and/or motor impairment (MCBMI) criteria,^74^ to distinguish those at different “pre‐symptomatic” stages. Finally, our study focused on integrating spatial maps of network activity in relation to atrophy and perfusion. Functional connectivity between networks is another important factor to be considered.^4^ The joint consideration of activity and connectivity might better characterize brain dynamics and cognitive performance.^75^ Future research could investigate the intercorrelations between functional connectivity and multiple neuroimaging modalities.

In conclusion, we demonstrated that frontoparietal network integrity might support cognitive function in pre‐symptomatic FTD. Linking neuroimaging, especially functional network integrity, with other neuropathological changes may be a future study direction for pre‐symptomatic genetic FTD. The dissociation of changes in structure, perfusion, and network activity in pre‐symptomatic FTD has implications for strategies to prevent or treat cognitive decline in people at high risk of FTD.

## CONFLICT OF INTEREST STATEMENT

James B. Rowe is a non‐remunerated trustee of the Guarantors of Brain, Darwin College, and the PSP Association; he provides consultancy to Alzheimer Research UK, Asceneuron, Alector, Biogen, CuraSen, CumulusNeuro, UCB, SV Health, and Wave, and has research grants from AZ‐Medimmune, Janssen, Lilly as industry partners in the Dementias Platform UK. All other authors have no conflict of interest to disclose. Author disclosures are available in the Supporting information.

## CONSENT STATEMENT

All participants provided informed consent.

## The GENFI Consortium Author List

Rhian Convery: Department of Neurodegenerative Disease, Dementia Research Centre, UCL Queen Square Institute of Neurology, London, UK. Martina Bocchetta: Department of Neurodegenerative Disease, Dementia Research Centre, UCL Queen Square Institute of Neurology, London, UK. David Cash: Department of Neurodegenerative Disease, Dementia Research Centre, UCL Queen Square Institute of Neurology, London, UK. Sophie Goldsmith: Department of Neurodegenerative Disease, Dementia Research Centre, UCL Queen Square Institute of Neurology, London, UK. Kiran Samra: Department of Neurodegenerative Disease, Dementia Research Centre, UCL Queen Square Institute of Neurology, London, UK. David L. Thomas: Neuroimaging Analysis Centre, Department of Brain Repair and Rehabilitation, UCL Institute of Neurology, Queen Square, London, UK. Thomas Cope: Cambridge University Hospitals NHS Trust, University of Cambridge, Cambridge, UK. Timothy Rittman: Department of Clinical Neurosciences, University of Cambridge, Cambridge, UK. Antonella Alberici: Centre for Neurodegenerative Disorders, Department of Clinical and Experimental Sciences, University of Brescia, Brescia, Italy. Enrico Premi: Stroke Unit, ASST Brescia Hospital, Brescia, Italy. Roberto Gasparotti: Neuroradiology Unit, University of Brescia, Brescia, Italy. Emanuele Buratti: ICGEB Trieste, Italy. Valentina Cantoni: Centre for Neurodegenerative Disorders, Department of Clinical and Experimental Sciences, University of Brescia, Brescia, Italy. Andrea Arighi: Fondazione IRCCS Ca’ Granda Ospedale Maggiore Policlinico, Neurodegenerative Diseases Unit, Milan, Italy. Chiara Fenoglio: University of Milan, Centro Dino Ferrari, Milan, Italy. Vittoria Borracci: Fondazione IRCCS Ca’ Granda Ospedale Maggiore Policlinico, Neurodegenerative Diseases Unit, Milan, Italy. Maria Serpente: Fondazione IRCCS Ca’ Granda Ospedale Maggiore Policlinico, Neurodegenerative Diseases Unit, Milan, Italy. Tiziana Carandini: Fondazione IRCCS Ca’ Granda Ospedale Maggiore Policlinico, Neurodegenerative Diseases Unit, Milan, Italy. Emanuela Rotondo: Fondazione IRCCS Ca’ Granda Ospedale Maggiore Policlinico, Neurodegenerative Diseases Unit, Milan, Italy. Giacomina Rossi: Fondazione IRCCS Istituto Neurologico Carlo Besta, Milano, Italy. Giorgio Giaccone: Fondazione IRCCS Istituto Neurologico Carlo Besta, Milano, Italy. Giuseppe Di Fede: Fondazione IRCCS Istituto Neurologico Carlo Besta, Milano, Italy. Paola Caroppo: Fondazione IRCCS Istituto Neurologico Carlo Besta, Milano, Italy. Sara Prioni: Fondazione IRCCS Istituto Neurologico Carlo Besta, Milano, Italy. Veronica Redaelli: Fondazione IRCCS Istituto Neurologico Carlo Besta, Milano, Italy. David Tang‐Wai: The University Health Network, Krembil Research Institute, Toronto, Canada. Ekaterina Rogaeva: Tanz Centre for Research in Neurodegenerative Diseases, University of Toronto, Toronto, Canada. Miguel Castelo‐Branco: Faculty of Medicine, ICNAS, CIBIT, University of Coimbra, Coimbra, Portugal. Morris Freedman: Baycrest Health Sciences, Rotman Research Institute, University of Toronto, Toronto, Canada. Ron Keren: The University Health Network, Toronto Rehabilitation Institute, Toronto, Canada. Sandra Black: Sunnybrook Health Sciences Centre, Sunnybrook Research Institute, University of Toronto, Toronto, Canada. Sara Mitchell: Sunnybrook Health Sciences Centre, Sunnybrook Research Institute, University of Toronto, Toronto, Canada. Christen Shoesmith: Department of Clinical Neurological Sciences, University of Western Ontario, London, Ontario, Canada. Robart Bartha: Department of Medical Biophysics, The University of Western Ontario, London, Ontario, Canada; Centre for Functional and Metabolic Mapping, Robarts Research Institute, The University of Western Ontario, London, Ontario, Canada. Rosa Rademakers: Center for Molecular Neurology, University of Antwerp. Jackie Poos: Department of Neurology, Erasmus Medical Center, Rotterdam, Netherlands. Janne M. Papma: Department of Neurology, Erasmus Medical Center, Rotterdam, Netherlands. Lucia Giannini: Department of Neurology, Erasmus Medical Center, Rotterdam, Netherlands. Liset de Boer: Department of Neurology, Erasmus Medical Center, Rotterdam, Netherlands. Julie de Houwer: Department of Neurology, Erasmus Medical Center, Rotterdam, Netherlands. Rick van Minkelen: Department of Clinical Genetics, Erasmus Medical Center, Rotterdam, Netherlands. Yolande Pijnenburg: Amsterdam University Medical Centre, Amsterdam VUmc, Amsterdam, Netherlands. Benedetta Nacmias: Department of Neuroscience, Psychology, Drug Research and Child Health, University of Florence, Florence, Italy. Camilla Ferrari: Department of Neuroscience, Psychology, Drug Research and Child Health, University of Florence, Florence, Italy. Cristina Polito: Department of Biomedical, Experimental and Clinical Sciences “Mario Serio”, Nuclear Medicine Unit, University of Florence, Florence, Italy. Gemma Lombardi: Department of Neuroscience, Psychology, Drug Research and Child Health, University of Florence, Florence, Italy. Valentina Bessi: Department of Neuroscience, Psychology, Drug Research and Child Health, University of Florence, Florence, Italy. Mattias Nilsson: Department of Clinical Neuroscience, Karolinska Institutet, Stockholm, Sweden. Henrik Viklund: Karolinska University Hospital Huddinge. Melissa Taheri Rydell: Department of Neurobiology, Care Sciences and Society; Center for Alzheimer Research, Division of Neurogeriatrics, Bioclinicum, Karolinska Institutet, Solna, Sweden; Unit for Hereditary Dementias, Theme inflammation and Aging, Karolinska University Hospital, Solna, Sweden. Vesna Jelic: Department of Neurobiology, Care Sciences and Society; Division of Clinical Geriatrics, Karolinska Institutet, Stockholm, Sweden; Cognitive clinic, Theme inflammation and Aging, Karolinska University Hospital, Solna, Sweden. Linn Öijerstedt: Department of Neurobiology, Care Sciences and Society; Center for Alzheimer Research, Division of Neurogeriatrics, Bioclinicum, Karolinska Institutet, Solna, Sweden; Unit for Hereditary Dementias, Theme inflammation and Aging, Karolinska University Hospital, Solna, Sweden. Tobias Langheinrich: Division of Neuroscience and Experimental Psychology, Wolfson Molecular Imaging Centre, University of Manchester, Manchester, UK; Manchester Centre for Clinical Neurosciences, Department of Neurology, Salford Royal NHS Foundation Trust, Manchester, UK. Albert Lladó: Alzheimer's disease and Other Cognitive Disorders Unit, Neurology Service, Hospital Clínic, Barcelona, Spain. Anna Antonell: Alzheimer's disease and Other Cognitive Disorders Unit, Neurology Service, Hospital Clínic, Barcelona, Spain. Jaume Olives: Alzheimer's disease and Other Cognitive Disorders Unit, Neurology Service, Hospital Clínic, Barcelona, Spain. Mircea Balasa: Alzheimer's disease and Other Cognitive Disorders Unit, Neurology Service, Hospital Clínic, Barcelona, Spain. Nuria Bargalló: Imaging Diagnostic Center, Hospital Clínic, Barcelona, Spain. Sergi Borrego‐Ecija: Alzheimer's disease and Other Cognitive Disorders Unit, Neurology Service, Hospital Clínic, Barcelona, Spain. Ana Verdelho: Department of Neurosciences and Mental Health, Centro Hospitalar Lisboa Norte – Hospital de Santa Maria & Faculty of Medicine, University of Lisbon, Lisbon, Portugal. Carolina Maruta: Laboratory of Language Research, Centro de Estudos Egas Moniz, Faculty of Medicine, University of Lisbon, Lisbon, Portugal. Tiago Coelho: Faculty of Medicine, University of Lisbon, Lisbon, Portugal. Gabriel Miltenberger: Faculty of Medicine, University of Lisbon, Lisbon, Portugal. Frederico Simões do Couto: Faculdade de Medicina, Universidade Católica Portuguesa. Alazne Gabilondo: Cognitive Disorders Unit, Department of Neurology, Donostia University Hospital, San Sebastian, Gipuzkoa, Spain; Neuroscience Area, Biodonostia Health Research Insitute, San Sebastian, Gipuzkoa, Spain. Ioana Croitoru: Neuroscience Area, Biodonostia Health Research Insitute, San Sebastian, Gipuzkoa, Spain. Mikel Tainta: Neuroscience Area, Biodonostia Health Research Insitute, San Sebastian, Gipuzkoa, Spain. Myriam Barandiaran: Cognitive Disorders Unit, Department of Neurology, Donostia University Hospital, San Sebastian, Gipuzkoa, Spain; Neuroscience Area, Biodonostia Health Research Insitute, San Sebastian, Gipuzkoa, Spain. Patricia Alves: Neuroscience Area, Biodonostia Health Research Insitute, San Sebastian, Gipuzkoa, Spain; Department of Educational Psychology and Psychobiology, Faculty of Education, International University of La Rioja, Logroño, Spain. Benjamin Bender: Department of Diagnostic and Interventional Neuroradiology, University of Tübingen, Tübingen, Germany. David Mengel: Department of Neurodegenerative Diseases, Hertie‐Institute for Clinical Brain Research and Center of Neurology, University of Tübingen, Tübingen, Germany; Center for Neurodegenerative Diseases (DZNE), Tübingen, Germany. Lisa Graf: Department of Neurodegenerative Diseases, Hertie‐Institute for Clinical Brain Research and Center of Neurology, University of Tübingen, Tübingen, Germany. Annick Vogels: Department of Human Genetics, KU Leuven, Leuven, Belgium. Mathieu Vandenbulcke: Geriatric Psychiatry Service, University Hospitals Leuven, Belgium; Neuropsychiatry, Department of Neurosciences, KU Leuven, Leuven, Belgium. Philip Van Damme: Neurology Service, University Hospitals Leuven, Belgium; Laboratory for Neurobiology, VIB‐KU Leuven Centre for Brain Research, Leuven, Belgium. Rose Bruffaerts: Department of Biomedical Sciences, University of Antwerp, Antwerp, Belgium; Biomedical Research Institute, Hasselt University, 3500 Hasselt, Belgium. Koen Poesen: Laboratory for Molecular Neurobiomarker Research, KU Leuven, Leuven, Belgium. Pedro Rosa‐Neto: Translational Neuroimaging Laboratory, McGill Centre for Studies in Aging, McGill University, Montreal, Québec, Canada. Maxime Montembault: Douglas Research Centre, Department of Psychiatry, McGill University, Montreal, Québec, Canada. Agnès Camuzat: Sorbonne Université, Paris Brain Institute – Institut du Cerveau – ICM, Inserm U1127, CNRS UMR 7225, AP‐HP – Hôpital Pitié‐Salpêtrière, Paris, France. Alexis Brice: Sorbonne Université, Paris Brain Institute – Institut du Cerveau – ICM, Inserm U1127, CNRS UMR 7225, AP‐HP – Hôpital Pitié‐Salpêtrière, Paris, France; Reference Network for Rare Neurological Diseases (ERN‐RND). Anne Bertrand: Sorbonne Université, Paris Brain Institute – Institut du Cerveau – ICM, Inserm U1127, CNRS UMR 7225, AP‐HP – Hôpital Pitié‐Salpêtrière, Paris, France; Inria, Aramis Project‐Team, F‐75013, Paris, France; Centre pour l'Acquisition et le Traitement des Images, Institut du Cerveau et la Moelle, Paris, France. Aurélie Funkiewiez: Sorbonne Université, Paris Brain Institute – Institut du Cerveau – ICM, Inserm U1127, CNRS UMR 7225, AP‐HP – Hôpital Pitié‐Salpêtrière, Paris, France; Centre de référence des démences rares ou précoces, IM2A, Département de Neurologie, AP‐HP – Hôpital Pitié‐Salpêtrière, Paris, France. Daisy Rinaldi: Sorbonne Université, Paris Brain Institute – Institut du Cerveau – ICM, Inserm U1127, CNRS UMR 7225, AP‐HP – Hôpital Pitié‐Salpêtrière, Paris, France; Centre de référence des démences rares ou précoces, IM2A, Département de Neurologie, AP‐HP – Hôpital Pitié‐Salpêtrière, Paris, France; Département de Neurologie, AP‐HP – Hôpital Pitié‐Salpêtrière, Paris, France. Dario Saracino: Sorbonne Université, Paris Brain Institute – Institut du Cerveau – ICM, Inserm U1127, CNRS UMR 7225, AP‐HP – Hôpital Pitié‐Salpêtrière, Paris, France; Centre de référence des démences rares ou précoces, IM2A, Département de Neurologie, AP‐HP – Hôpital Pitié‐Salpêtrière, Paris, France; Inria, Aramis project‐team, F‐75013, Paris, France. Olivier Colliot: Sorbonne Université, Paris Brain Institute – Institut du Cerveau – ICM, Inserm U1127, CNRS UMR 7225, AP‐HP – Hôpital Pitié‐Salpêtrière, Paris, France; Centre pour l'Acquisition et le Traitement des Images, Institut du Cerveau et la Moelle, Paris, France; Inria, Aramis project‐team, F‐75013, Paris, France. Sabrina Sayah: Sorbonne Université, Paris Brain Institute – Institut du Cerveau – ICM, Inserm U1127, CNRS UMR 7225, AP‐HP – Hôpital Pitié‐Salpêtrière, Paris, France. Catharina Prix: Neurologische Klinik, Ludwig‐Maximilians‐Universität München, Munich, Germany. Elisabeth Wlasich: Neurologische Klinik, Ludwig‐Maximilians‐Universität München, Munich, Germany. Olivia Wagemann: Neurologische Klinik, Ludwig‐Maximilians‐Universität München, Munich, Germany. Sonja Schönecker: Neurologische Klinik, Ludwig‐Maximilians‐Universität München, Munich, Germany. Alexander Maximilian Bernhardt: Neurologische Klinik, Ludwig‐Maximilians‐Universität München, Munich, Germany. Anna Stockbauer: Neurologische Klinik, Ludwig‐Maximilians‐Universität München, Munich, Germany. Jolina Lombardi: Department of Neurology, University of Ulm, Ulm. Sarah Anderl‐Straub: Department of Neurology, University of Ulm, Ulm, Germany. Adeline Rollin: CHU, CNR‐MAJ, Labex Distalz, LiCEND Lille, France. Gregory Kuchcinski: Univ Lille, France; Inserm 1172, Lille, France; CHU, CNR‐MAJ, Labex Distalz, LiCEND Lille, France. Maxime Bertoux: Inserm 1172, Lille, France; CHU, CNR‐MAJ, Labex Distalz, LiCEND Lille, France. Thibaud Lebouvier: Univ Lille, France; Inserm 1172, Lille, France; CHU, CNR‐MAJ, Labex Distalz, LiCEND Lille, France. Vincent Deramecourt: Univ Lille, France; Inserm 1172, Lille, France; CHU, CNR‐MAJ, Labex Distalz, LiCEND Lille, France. João Durães: Neurology Department, Centro Hospitalar e Universitario de Coimbra, Coimbra, Portugal. Marisa Lima: Neurology Department, Centro Hospitalar e Universitario de Coimbra, Coimbra, Portugal. Maria João Leitão: Centre of Neurosciences and Cell Biology, Universidade de Coimbra, Coimbra, Portugal. Maria Rosario Almeida: Faculty of Medicine, University of Coimbra, Coimbra, Portugal. Miguel Tábuas‐Pereira: Neurology Department, Centro Hospitalar e Universitario de Coimbra, Coimbra, Portugal; Faculty of Medicine, University of Coimbra, Coimbra, Portugal. Sónia Afonso: Instituto Ciencias Nucleares Aplicadas a Saude, Universidade de Coimbra, Coimbra, Portugal. João Lemos: Faculty of Medicine, University of Coimbra, Coimbra, Portugal.

## Supporting information



Supporting Information

Supporting Information
